# Role of Exosomal Noncoding RNAs in Lung Carcinogenesis

**DOI:** 10.1155/2015/125807

**Published:** 2015-10-25

**Authors:** Tao Sun, Bill Kalionis, Guoying Lv, Shijin Xia, Wen Gao

**Affiliations:** ^1^Shanghai Institute of Geriatrics, Huadong Hospital, Fudan University, Shanghai 200040, China; ^2^Department of Perinatal Medicine, Pregnancy Research Centre and University of Melbourne Department of Obstetrics and Gynaecology, Royal Women's Hospital, Parkville, VIC 3052, Australia; ^3^Emergency Department of Wuhan Hospital of Traditional Chinese Medicine, Wuhan 430010, China; ^4^Department of Thoracic Surgery, Huadong Hospital, Fudan University, Shanghai 200040, China

## Abstract

Lung cancer is the major cause of cancer death worldwide. Novel, recently discovered classes of noncoding RNAs (ncRNAs) have diverse functional and regulatory activities and increasing evidence suggests crucial roles for deregulated ncRNAs in the onset and progression of cancer, including lung cancer. Exosomes are small extracellular membrane vesicles of endocytic origin that are released by many cells and are found in most body fluids. Tumor-derived exosomes mediate tumorigenesis by facilitating tumor growth and metastasis. MicroRNAs (miRNAs) are a subclass of ncRNAs that are present in exosomes. miRNAs are taken up by neighboring or distant cells and modulate various functions of recipient cells. Here, we review exosome-derived ncRNAs with a focus on miRNAs and their role in lung cancer biology.

## 1. Introduction

Lung cancer is one of the most lethal human cancers worldwide. Nonsmall cell lung cancers (NSCLC) are the majority of lung cancers (80%) while the remaining are small cell lung cancers (SCLC) [1]. Despite recent advances in lung cancer research and the use of novel therapeutic agents for NSCLC, outcomes remain dismal with a 5-year survival rate of about 15% [1]. A deeper understanding of the molecular mechanisms underlying NSCLC development and progression is needed.

ncRNAs are a novel class of RNA molecules that perform many basic regulatory functions in eukaryotes and these include regulating normal cell growth and apoptosis but they also play roles in abnormal cell functions associated with cancer progression and metastasis [2, 3]. However, the functional role of ncRNAs in NSCLC is not well understood. ncRNAs are a large class of molecules that can be divided into two groups based on their transcript lengths: small ncRNAs (<200 bp), such as transfer RNAs (tRNAs), ribosomal RNAs (rRNAs), small nucleolar RNAs (snoRNAs), microRNAs (miRNAs), small interfering RNAs (siRNA), PIWI-interacting RNAs (piRNA), antisense RNAs, and promoter-associated RNAs (PARs) and long ncRNAs (lncRNAs, >200 bp) [4].

Cell-cell communication can occur by several means, including chemical receptor-mediated events, direct cell-cell contact and cell-cell synapses. Extracellular vesicles (EVs), which include exosomes and microvesicles, are mediators of cell-cell communication and are novel biomarkers for disease. Increasing evidence implicates cell-to-cell communication, which can be mediated by delivery of miRNAs after transfer through exosomes, in physiological and pathological processes. Exosomes are subcellular vesicles (30–120 nm in diameter) present in biological fluids. Different miRNAs including let-7, miR-1, miR-15, miR-16, miR-181, and miR-375, which seem to be specifically packaged exclusively into exosomes [5], have sparked intense investigations into the composition and function of exosomes. miRNAs are small ncRNA gene products discovered in diverse organisms that are thought to regulate target gene expression [6]. A potential role for miRNAs in carcinogenesis comes from their ability to inhibit the translation of tumor suppressor genes and oncogenes. Many human cancers display unique miRNA expression signatures. miRNAs can be secreted via extracellular vesicles (EVs) and by protein-miRNA complexes. Current studies show that miRNAs are present in extracellular spaces, packaged into various membrane-bound vesicles. The protein-miRNA content of EVs can be transferred to recipient cells and deliver biological information that plays an important role in cancer metastasis and prognosis.

Exosomal miRNAs are also body fluid miRNAs but they differ from free miRNAs circulating in the body fluid because they are encapsulated in exosomes derived from the multivesicular body (MVB) sorting pathway [7]. Exosomes are distributed in various body fluids and are taken up by neighboring or distant cells. In doing so, miRNAs are delivered to the recipient cell and then they alter cell functions. Ratajczak et al. showed RNA contained within exosomes was responsible for the horizontal transfer of genetic information between cells [8]. Ribonuclease pretreatment inhibited the functional effects of exosomes, which provided convincing evidence of RNA involvement. Following translocation from the nucleus to the cytoplasm, RNA molecules bind to membranous organelles or vesicles and are transported to intracellular sites, which may account for RNA populations associating with exosomes. Valadi et al. showed that exosomes contain a subset of both cellular mRNA and miRNA, which could be transferred to recipient cells [5].

Many studies show that miRNAs are differentially enriched in normal exosomes compared with exosomes from pathological conditions such as tumors. Exosome-containing miRNAs are implicated in cancer pathogenesis [9]. For example, microarray analysis revealed selective enrichment of let-7 miRNA family in exosomes derived from highly metastatic gastric cancer cells, when compared with lowly metastatic gastric cells [10]. Members of the let-7 miRNA family have tumour suppressor activity that targets oncogenes such as RAS and HMGA2. Ohshima et al. [10] proposed a mechanism where highly invasive tumour cells release let-7 miRNAs through exosomes into the extracellular milieu, to maintain their tumourigenic phenotype.

Cancer cell communication is an important and complex process, which is achieved through diverse mechanisms and allows tumor cells to mold and influence their environment. Recent evidence shows that cells communicate via the release and delivery of miRNAs packed into tumor-released (TR) exosomes. Some miRNAs are either over- or underexpressed in particular cancer types. Patterns of miRNAs are unique to individual tissue types and differ between cancer and normal samples [11, 12]. Current data support miRNAs as potential biomarkers for NSCLC [13–17]. Measurement of levels of multiple miRNAs can more precisely gauge prognosis and accurately predict survival in lung cancer [18].

## 2. The Release Mechanism of Circulating miRNAs

The biological mechanisms that culminate in the release of circulating miRNAs remain unclear. Possible mechanisms for miRNA release include (1) active secretion of miRNAs as vesicle-containing miRNAs and (2) energy-free passive leakage of cellular miRNAs from disrupted cells. The latter mechanism is only a minor contributor to the process of circulating miRNA generation and occurs predominantly under pathological circumstances such as tissue damage, cell apoptosis, tumor metastatic process, or chronic inflammation [19, 20]. Circulating miRNAs are packaged into various membrane-bound vesicles including exosomes and microvesicles [5], whereas vesicle-free miRNAs are associated with protein or high-density lipoprotein complexes [21]. Only EV-derived miRNAs function in cell-cell communication, and they play roles in various biological processes including immune system regulation, inflammation, and tumor development [22, 23]. EV-derived miRNAs control many aspects of human physiological status and are therefore potentially better disease biomarkers than other circulating miRNAs.

Unfortunately, the release mechanism of circulating miRNAs by tumors is not well established. Rabinowits et al. evaluated the exosomal miRNA circulating levels of patients with lung adenocarcinoma and compared them with those of patients without lung cancer, showing that the miRNA signatures of exosomes parallel those of the miRNA expression profiles of the originating tumor cells. This indicates that the content of tumor cell-derived exosomes is correlated with the miRNA levels in primary tumor [24]. Fabbri et al. provided evidence that, in immune cells, tumor-secreted miR-21 and miR-29a act as ligands that bind receptors of the toll-like receptor (TLR) family, specifically human TLR8, and murine TLR7. Tumor growth and metastasis would be a consequence miRNA binding to TLR that mediates a prometastatic inflammatory response [9].

## 3. Potential Modes for Sorting of miRNAs into Exosomes

**Figure 1:**
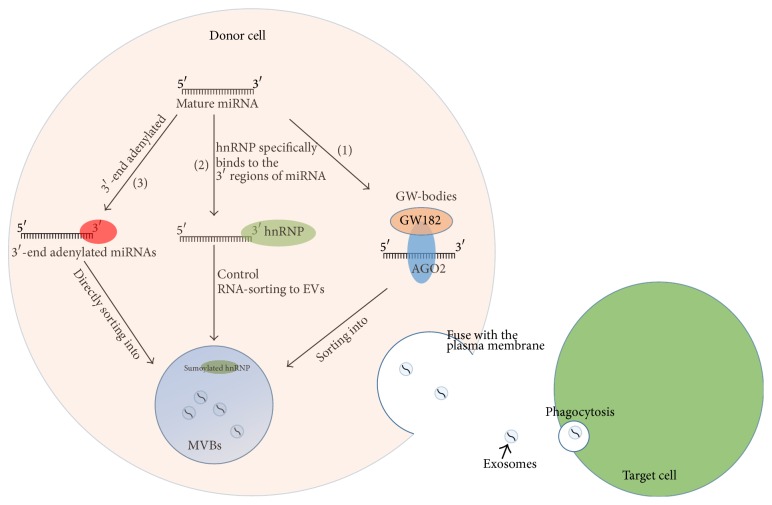
Potential modes for sorting of miRNAs into exosomes: (1) Mature miRNAs can interact with assembly proteins to form a complex called miRISC, which includes target miRNA, miRNA-repressible mRNA, GW182 protein, and miRNA effector protein argonaute 2 (AGO2, which was initially identified as membrane associated). Active miRISCs are recruited into GW-bodies that are physically associated with MVBs and this may represent a method of loading miRNAs into exosomes. (2) The protein hnRNP specifically binds to the 3′ regions of miRNA sequences and controls their loading into exosomes. (3) 3′-end adenylated miRNAs directly sort into EVs. miRNAs release as exosomes and uptake by recipient cell: (a) MVBs are released by exocytosis and fuse with the plasma membrane leading to the release of exosomes. (b) EVs uptake by recipient cells through phagocytosis.

Current research describes four possible modes for sorting of the specific loading of miRNA species into exosomes, although the underlying mechanisms are unclear (Figure 1).

The first mode of sorting involves the miRNA-induced silencing complex- (miRISC-) related pathway. Mature miRNAs can interact with assembly proteins to form a complex called miRISC, which includes miRNA, miRNA-repressible mRNA, GW182 protein, and miRNA effector protein argonaute 2 (AGO2). Multivesicular bodies (MVBs, also called late endosomes) are derived from early endosomes. MVBs either release exosomes into the extracellular space upon fusion with plasma membrane or fuse with lysosomes and degrade their content. GW-bodies are distinct foci within the eukaryotic cell cytoplasm that participate in various types of mRNA decay and miRNA-induced mRNA silencing. Gibbings et al. showed GW-bodies appear to contain most of the miRISC complex components (including GW182 protein and AGO2), colocalise with MVBs, and may be sites where miRISCs accumulate and act. They also showed that exosome-like vesicles produced by MVBs were enriched in GW182 protein but not AGO2. Furthermore, blocking the formation of MVB by reducing endosomal sorting complex, required for transport (ESCRT) components, reduced miRNA-mediated gene silencing and resulted in excess accumulation of GW182 protein, while preventing the fusion of MVBs with lysosomes stimulated RISC activity. These data are consistent with the idea that active miRISCs with target mRNAs are recruited into GW-bodies that are physically associated with MVBs and this may represent a method of loading miRNAs into exosomes [25]. The second mode involves a miRNA motif and the sumoylated heterogeneous nuclear ribonucleoproteins- (hnRNPs-) dependent pathway. The protein hnRNPA2B1 specifically binds exosomal miRNAs through the recognition of these motifs in the 3′ regions of miRNA sequences and controls their loading into exosomes. In addition, hnRNPA2B1 in exosomes is preferentially sumoylated, and this sumoylation is important for the loading of exosomal miRNAs into exosomes. The loading of miRNAs into exosomes can be modulated by mutagenesis of the identified motifs or changes in hnRNPA2B1 expression levels [26]. Another two hnRNP family proteins, hnRNPA1 and hnRNPC, could also bind to exosomal miRNAs, suggesting that they might also be candidates for miRNA sorting [27]. The third mode relies on a pathway dependent on the 3′ ends of the miRNA. 3′-end adenylated miRNAs are relatively enriched in cells, whereas 3′-end uridylated isoforms appear overrepresented in exosomes derived from B cells or urine. Collectively, it suggests that posttranscriptional modifications, notably 3′-end adenylation and uridylation, exert opposing effects that may contribute to direct ncRNA sorting into EVs [27]. Therefore, two sorting modes show that the 3′ regions or the 3′ ends of the miRNA sequence contain as yet unidentified critical sorting signal(s). The last mode is mediated by the neural sphingomyelinase 2- (nSMase2-) dependent pathway. Kosaka et al. demonstrate that nSMase2 regulates exosomal miRNA secretion and promotes angiogenesis within the tumor microenvironment as well as metastasis. nSMase2 is the initial molecule associated with miRNA secretion into exosomes. Overexpressed nSMase2 increased the number of exosomal miRNAs whereas inhibition of nSMase2 expression had the converse effect [28].

There are two ways for microvesicles secreting from donor cells: (1) microvesicles are directly shed from the cell membrane; and (2) MVBs which are released by exocytosis fuse with the plasma membrane and release the intraluminal endosomal vesicles into the extracellular space to become exosomes [29]. The miRNAs in cell-released exosomes can circulate with the associated vehicles to reach neighboring cells and distant cells. EVs, which interact with the functionally active receptors (CCR5, EGFRvIII, or MET) or ligands of target cells, may induce changes in the cell phenotype, eventually leading to triggering intracellular signaling pathways [30]. EVs uptake by recipient cells seems to occur through phagocytosis [31]. After being delivered into target cells, exosomal miRNAs play functional roles (Figure 1).

## 4. The ncRNAs Related to Lung Cancer

Great progress in the last decade has increased our understanding of the role miRNAs play in lung cancer. Several miRNAs that display different patterns in lung cancer compared to normal tissues were identified. Aberrant miRNA levels were detected in lung cancer tumor tissues but also in body fluids and extracellular organelles, such as exosomes. These combined studies gave weight to the notion that miRNAs are promising biomarkers for improved diagnosis and prediction, and they are potential targets for therapeutics to treat lung cancer.

Cazzoli et al. screened 742 miRNAs in circulating exosomes and identified four miRNAs (miR-378a, miR-379, miR-139-5p, and miR-200b-5p) as screening markers to segregate lung adenocarcinoma and carcinoma patients, from healthy former smokers. They also identified six miRNAs (miR-151a-5p, miR-30a-3p, miR-200b-5p, miR-629, miR-100, and miR-154-3p) that segregated lung adenocarcinoma patients and lung granuloma patients [32].

In 2004, Takamizawa et al. [33] identified the first miRNA family, let-7, which was associated with the tumorigenesis of lung cancer. They found that introduction of let-7a and let-7f isoforms into a lung adenocarcinoma cell line with low baseline levels of let-7 expression (A549), inhibited the growth of A549 cells. In a clinical setting, reduced let-7 levels were associated with shorter patient survival after diagnosis. Many targets of let-7 are now known, including the RAS family [34], HMGA2 [34–36], c-Myc [37, 38], CDC25A, CDK6, and Cyclin D2 [39]. These target genes helped to reveal the mechanisms by which let-7 family members exert their function in tumorigenesis. Many miRNAs are oncogenes or tumor suppressor genes and these include miR-17–92 [16, 40], miR-218 [41], miR-21 [42], and miR-34 family members (miR-34a and miR-34b/c) [43–47]. miRNAs play pivotal roles in lung tumorigenesis and are also involved in tumor metastasis. Several miRNAs including miR-17–92 [48–51], the miR-200 family of miRNAs (miR-200a, miR-200b, miR-200c, miR-141, and miR-429) [52], miR-125a-3p/5p [53], miR-21 [54], and the miR-106b-25 cluster (miR-106b and miR-93) [55] are implicated in metastatic lung cancer. Many other miRNAs and their potential mechanism(s) of action are shown in Table 1.

Besides miRNAs, long ncRNAs (lncRNAs) with tumour-promoting and tumour-suppressing functions in lung cancer have also been identified [56]. MALAT1 is lncRNA present at high levels, which is upregulated during metastasis of early-stage NSCLC and is a powerful predictor of metastatic relapse in patients with NSCLC. SCAL1 is lncRNA that shows increased levels as part of the oxidative stress response of airway epithelial cells to cigarette smoke. SCAL1 is also overexpressed in a range of lung cancer cell lines, which may indicate a novel mechanism of smoke-induced malignant transformation. Yang et al. characterized the function of lncRNA PVT1 in NSCLC development and progression [57]. PVT1 levels were increased in NSCLC tissues and lung cancer cell lines when compared with matching nearby normal tissues and the normal human bronchial epithelial cell line 16HBE, respectively. They also found PVT1 levels correlated positively with histological grade and lymph node metastasis, but there was no correlation with age, gender, and tumor size in patients with NSCLC. High levels of PVT1 correlated with lower overall survival rates and were an independent prognostic factor in patients with NSCLC. These results provided evidence of a potentially important role of PVT1 in tumorigenesis and in the progression of NSCLC. Other lncRNAs and their potential mechanism(s) of action are shown in Table 2.

## 5. Potential Mechanism of Some Exosomal miRNAs

Silva et al. showed levels of exosomal let-7f and/or miR-30e-3p in NSCLC patients could distinguish between patients with resectable and nonresectable tumors [58]. Rabinowits et al. assessed patients with and without lung adenocarcinoma for their levels of circulating tumor exosomes and exosomal small RNA as well as specific exosomal miRNAs. Subsequently, these levels were correlated with disease stages as defined by the American Joint Committee on Cancer (AJCC). These studies were used to validate markers for diagnosis and prognosis in patients for lung adenocarcinoma. The levels of 12 exosomal miRNAs (miR-17-3p, miR-21, miR-106a, miR-146, miR-155, miR-191, miR-192, miR-203, miR-205, miR-210, miR-212, and miR-214) were significantly different between patients and controls [24]. The potential mechanism(s) of those 12 exosomal miRNAs are shown in Table 1.

The analysis of specific miRNAs in exosomes showed enrichment of miRNAs in samples from patients with NSCLC both in plasma and in bronchoalveolar lavage. This finding, together with the higher proportion of exosomes detected in the plasma of NSCLC patients, supports the idea that a proliferative advantage accrues to tumors that release exosomes into plasma [59].

let-7 was the first miRNA shown to be dysregulated in human lung cancer [33]. Decreased levels of let-7 in the sputum of patients with chronic obstructive pulmonary disease (COPD) fueled speculation that chronic airway inflammation, combined with the loss of a major tumour suppressor, may contribute to the increased risk of patients with COPD developing lung cancer. Yu et al. identified a five-miRNA signature that can predict survival in patients with lung cancer, of which hsa-miR-221 and hsa-let-7a were protective, while hsa-miR-137, hsa-miR-372, and hsa-miR-182^*∗*^ were nonprotective [18]. In patients with lung cancer, the total levels of exosomes and their let-7 miRNAs increased compared to controls. Ohshima et al. performed extensive miRNA analysis in three cellular fractions including cells, exosomes, and medium from cultured cells and they found that let-7 miRNA family is rich in all the fractions from the gastric cancer cell line, AZ-P7a. After comparing the levels of let-7 miRNA family members in exosomes derived from AZ-P7a with exosomes from other cancer cell lines, including the lung cancer cell lines (SBC-3, DMS-35, and NCI-H69), they found members of the let-7 miRNA family were abundant in exosomes derived from AZ-P7a but were less abundant in exosomes derived from other cancer cells. These data were consistent with the notion that exosome-mediated release of let-7 miRNAs into the extracellular milieu resulted in a reduced antitumorigenic effect within the cells and consequently oncogenesis and invasiveness of the cells were sustained [10]. Levels of let-7f, miR-20b, and miR-30e-3p in vesicles from the plasma of NSCLC patients were lower than normal controls [58]. The let-7 family members negatively regulate the oncogene RAS [34], suggesting that let-7 acts biologically as a tumor suppressor.

The let-7 miRNA family members are important regulators that control lung cancer oncogene expression by binding to the 3′ untranslated regions (3′-UTR) of their target mRNAs, and lower levels of let-7 miRNA have been found in NSCLC [34, 102]. let-7 regulates many key cell cycle protooncogenes (KRAS, CDC25a, CDK6, c-MYC, cyclin D, and BCL-2), and in this way let-7 controls cell proliferation by negatively regulating the pathways promoting the G1 to S transition [39]. An interesting feature of the let-7 family is that the 3′-UTR of KRAS (as well as HRAS, NRAS, and various members of the RAS GTPase family) contain multiple let-7 binding sites, and let-7 levels in lung cancer inversely correlate with KRAS expression [103]. Chin et al. described a novel SNP (Single Nucleotide Polymorphism) in the 3′-UTR of the KRAS gene that influences let-7 binding to its target site. This variant, the let-7 complementary site (LCS6) in the KRAS 3′-UTR, is associated with KRAS gene upregulation and let-7 lower levels; however this polymorphism correlates with a modest increase in lung cancer risk [104]. A recent study identified HOXA1 as a new target of the let-7 family. Zhan et al. showed that let-7c represses NSCLC cell proliferation and tumorigenesis by targeting the 3′-UTR of HOXA1 mRNA, which reduced the expression of CCND1, CDC25A, and CDK2 [71]. He et al. showed that let-7a, a member of let-7 family, negatively regulated the expression of NIRF (Np95/ICBP90-like RING finger protein) through NIRF 3′ UTR. Enforced expression of let-7a in A549 lung cancer cells decreased NIRF leading to a coordinated increase in p21^WAF1^ [105]. NIRF binds to methyl-CpGs of the promoter region through a SET and RING finger-associated (SRA) domain. This protein constitutes a complex with HDAC1 (histone deacetylase-1) also via its SRA domain, which binds to methylated promoter regions of various tumor suppressor genes, including p21^WAF1^ in cancer cells [106]. Recently [107], let-7c levels were observed to be inversely correlated to levels of ITGB3 (integrin b3, also known as CD61) and MAP4K3, a member of the MAP4K family in NSCLC tissues.

Gao et al. identified miRNA profiles in NSCLC and showed that deregulated expression of miR-21, miR-143, and miR-181a correlated with patient prognosis, suggesting that miRNAs are involved in the initiation and progression of this disease [108]. miR-21 is the most common miRNA in the human lung but at elevated levels miR-21 contributes to the symptomatic development of asthma, idiopathic pulmonary fibrosis, and non-small-cell lung cancer (NSCLC) through its effects on inflammatory and cancer suppressing genes. miR-21 is an antiapoptotic miRNA and is regulated by the EGFR pathway and miR-21 levels correlate with phosphorylated EGFR levels [109]. miR-21 stimulates cell growth and NSCLC cell invasion by targeting PTEN (phosphatase and tensin homolog), which enhances the RAS/MEK/ERK pathway by repressing negative regulators of the pathway (i.e., Spry1, Spry2, Btg2, and Pdcd4) and by repressing the expression of proapoptotic proteins (e.g., Apaf1, FasL, RhoB, and Pdcd4). miR-21 is also upregulated by KRAS in NSCLC, both* in vitro* and* in vivo,* through MAPK/AP-1 activation [42, 109–111]. Chiba et al. revealed that exosomes derived from the colorectal cancer cell lines contain miR-21, miR-192, and miR-221 and can be delivered into lung cancer A549 cells [112].

miR-126 is a tumor suppressor, because of its antioncogenic properties. miR-126 inhibits NSCLC cell line proliferation through EGFL7 [113] and targets the Sdf-1a cytokine, which reduces mesenchymal stem cell and inflammatory monocyte recruitment to primary tumors, thereby inhibiting lung metastasis [114]. Rodríguez et al. showed that tumors may eliminate miR-126 by exosome liberation [59].

## 6. Conclusion

Exosomes, despite their nanosize, can act as key communication facilitators between cells. Gradually, we are revealing the roles of exosomes in interactions between distant cells and the complex roles of exosomes in tumorigenesis and cancer progression. Exosomes secrete a variety of biological molecules, including miRNAs, proteins, and their complexes. The study of exosomal miRNAs offers a new and exciting approach to understanding the molecular mechanisms of lung cancer biology. However, the study of exosomes in lung carcinogenesis is still in its infancy. Unresolved issues include whether other noncoding RNAs such as lncRNAs are present in exosomes and whether lncRNAs are involved in target gene regulation in recipient cells. Much work remains to be carried out before we have a complete understanding of the role of exosomal ncRNAs in lung carcinogenesis.

## Figures and Tables

**Table 1:** The potential mechanisms and target genes of miRNAs in lung cancer.

Onco-miRNA	Potential mechanisms	Target genes	Reference
miR-21	Overexpression of miR-21 enhances tumorigenesis and inhibits apoptosis through inhibition of negative regulators of the Ras/MEK/ERK pathway and inhibition of apoptosis	PTEN, SPRY1, SPRY2, BTG2, PDCD4, APAF1, FasL, and RHOB	[42]
miR-212	Exert an antiapoptotic effect through direct repression of synaptic acetylcholinesterase expression in NSCLC	AChE	[60]
miR-17–92 cluster	Enhances cell proliferation, inhibits apoptosis, and protects against DNA damage	p21, CTGF, Tsp1, PTEN, Bim, and HIF-1*α*	[16, 61]
miR-221-222	Activates the PI3K/Akt pathway and metallopeptidases	Kit, p27 kip1, PTEN/TIMP3, PUMA, and TRAIL	[62]
miR-93, miR-98, and miR-197	Inhibits tumor suppressor gene FUS1 expression	FUS1	[63]
miR-375	miR-375 upregulation and correlates with ASCL1 in the cell lines generated from mouse SCLC-like tumors	Not determined	[64]
miR-17-5p and miR-20a	miRNA inhibition reduces cell growth miRNA inhibition induces apoptosis and increases the proportions of sub-G1 populations	E2F1	[65]
miR-328	Associated with cell migration and NSCLC brain metastases by controlling the VEGF/IL1 signaling pathway	PRKCA, VEGF-D, NOTCH1, IL1-*α*, IL1-*β*, and PLC-*γ*	[66]
miR-106	Upregulated in lung cancer	RB	[67]
miR-155	Promotes cell proliferation through the repression of SOCS1 Downregulates several tumor suppressors such as PTEN, PDC4, and SHIP1, leading to the activation of the Akt pathway Plays a role in cell invasion by targeting RhoA	CASP3, TP53BP1, SOCS1, PTEN, PDC4, and SHIP1	[68–70]

Tumor suppressor miRNA	Potential mechanisms	Target genes	Reference

let-7 family	Suppress cell proliferation by negatively regulating pathways promoting the G1 to S transition	KRAS, CDC25a, CDK6, c-MYC, CCND1, and BCL-2	[39, 71]
miR-126	Inhibits cell proliferation by arresting the cells in the G1 phase by targeting VEGF	VEGF, CRK, and SLC7A5,	[49, 72]
miR-26a	Inhibits cell proliferation, blocks G1/S phase transition, induces apoptosis, and inhibits cell metastasis and invasion *in vitro *	EZH2	[73]
miR-7	Involved in miR-7-mediated growth suppression and apoptosis Inhibits cancer cell migration	BCL-2	[74]
miR-335	Reduces cell migration, invasion, proliferation, and metastasis	BCL-W and SP1	[75]
miR-145	Inhibits cell growth, proliferation, and migration of lung cancer cells and induces cell cycle arrest in G1 by targeting CDK4	EGFR, NUDT1, CDK4, c-Myc, and OCT4	[76–78]
miR-413	Inhibits cell proliferation and enhances apoptosis	PKC*ε*	[79]
miR-192	Inhibits cell proliferation and induces cell apoptosis in lung cancer cells Arrests cells in G1 phase	RB1	[80]
miR-449 cluster	Induces apoptosis targeting Bcl-2, n-MYC, and HDAC1 and upregulates p53 through the repression of deacetylase gene SIRT1 Inhibits cell migration and invasion by suppression of AXL and SNAIL-1	CDK4, CDK6, c-MYC, CCND1, CCNE2, CDC25A, MET, and E2F	[81–83]
miR-206	Overexpression of miR-206 inhibits migration and invasion of lung cancer cells	Not determined	[84]
miR-146a	Inhibits cell growth and induces cell apoptosis Suppresses motility Enhances cell proliferation inhibitory effect of TKIs and cetuximab	EGFR	[85]
miR-203	Inhibits cell proliferation and migration of lung cancer	PKC*α*	[86]
miR-205	Regulates epithelial to mesenchymal transition by targeting ZEB1 and SIP1	Not determined	[87]
miR-214	Regulates the acquired resistance to gefitinib via the PTEN/AKT pathway	PTEN	[88]

**Table 2:** The potential mechanisms of lncRNAs in lung cancer.

Onco-lncRNA	Potential mechanism	Reference
MVIH	Affects expression of MMP-2/MMP-9 Proliferation and invasion	[89]
BC200	Upregulated in lung cancer	[90]
lncRNA-DQ786227	Upregulated in lung cancer	[91]
MALAT1	Affects expression of Bcl-2 and metastasis related genes	[92]
AK126698	Decreases NKD and increases the accumulation and nuclear translocation of *β*-catenin Antiapoptotic effects and resistance to cisplatin	[93]
SCAL1	Induced by cigarette smoke and NRF2 Protective against oxidative stress	[94]
HOTAIR	Induced by Col-1 upregulation of cell adhesion-related genes such as ASTN1 and PCDHA1 Downregulates genes involved in neuronal growth and signal transduction including NTM and PTK2B Contributes to the cisplatin resistance through the regulation of p21 expression Affects proliferation, migration, and invasion	[95–97]

Tumor suppressor lncRNA	Potential mechanism	Reference

BANCR	A regulator of EMT during NSCLC Induces apoptosis and inhibits EMT, migration, invasion, and metastasis	[98]
MEG3	Inhibits NSCLC cell proliferation and induces apoptosis by affecting p53 expression	[99]
SPRY4-IT1	Promotes NSCLC cell proliferation and metastasis via effects on the epithelial-mesenchymal transition	[100]
TUG1	Affects cell proliferation in human NSCLC through epigenetically regulating HOXB7 expression	[101]
